# Butyrate Producers as Potential Next-Generation Probiotics: Safety Assessment of the Administration of *Butyricicoccus pullicaecorum* to Healthy Volunteers

**DOI:** 10.1128/mSystems.00094-18

**Published:** 2018-11-06

**Authors:** Leen Boesmans, Mireia Valles-Colomer, Jun Wang, Venessa Eeckhaut, Gwen Falony, Richard Ducatelle, Filip Van Immerseel, Jeroen Raes, Kristin Verbeke

**Affiliations:** a Translational Research Center for Gastrointestinal Disorders (TARGID), KU Leuven, Leuven, Belgium; b Laboratory of Molecular Bacteriology, Department of Microbiology and Immunology, Rega Institute, KU Leuven, Leuven, Belgium; c Center for Microbiology, VIB, Leuven, Belgium; d Department of Pathology, Bacteriology and Avian Diseases, Faculty of Veterinary Medicine, Ghent University, Merelbeke, Belgium; e Research Group of Microbiology, Department of Bioengineering Sciences, Vrije Universiteit Brussel, Brussels, Belgium; f Leuven Food Science and Nutrition Research Center, KU Leuven, Leuven, Belgium; Teagasc Food Research Centre

**Keywords:** *Butyricicoccus pullicaecorum*, metabolome, microbiome, next-generation probiotic, safety, tolerance

## Abstract

Advances in gut microbiota research have triggered interest in developing colon butyrate producers as niche-specific next-generation probiotics, targeted at increasing colon butyrate production and countering disease-associated microbiota alterations. Crucial steps in the development of next-generation probiotics are the design of formulations with a reasonable shelf life as well as the safety demonstration of an intervention in healthy volunteers. One such potential next-generation butyrate-producing probiotic is Butyricicoccus pullicaecorum 25-3^T^, with demonstrated safety in *in vitro* as well as animal models. Here, we examined the strain’s safety, tolerability, and impact on microbiota composition and metabolic activity in healthy volunteers in a randomized, double-blind, placebo-controlled crossover study in 30 healthy volunteers. The study design consisted of two 4-week intervention periods (10^8^ CFU B. pullicaecorum [treatment] or maltodextrin [placebo] per day) with a 3-week washout in between. We assessed adverse events, blood parameters (primary endpoints), and fecal microbiota composition and metabolite profiles (secondary endpoints). The number of reported adverse events during the B. pullicaecorum treatment was similar to that of placebo intervention, as were observed changes in blood chemistry parameters, bowel habits, and fecal calprotectin concentrations. Administration of the strain did not induce any disruptive effect in microbiota composition or metabolic activity. In this first human intervention trial with a butyrate-producing Clostridium cluster IV isolate, we demonstrated B. pullicaecorum 25-3^T^ administration to be both safe and well tolerated by healthy participants. This safety study paves the way for the further development of the strain as a next-generation probiotic.

**IMPORTANCE** This study is the first to determine the safety and tolerance in humans of a butyrate-producing Clostridium cluster IV next-generation probiotic. Advances in gut microbiota research have triggered interest in developing colon butyrate producers as next-generation probiotics. Butyricicoccus pullicaecorum 25-3^T^ is one such potential probiotic, with demonstrated safety *in vitro* as well as in animal models. Here, we produced an encapsulated B. pullicaecorum formulation that largely preserved its viability over an 8-month storage period at 4°C. Administration of this formulation to healthy volunteers allowed us to establish the intervention as safe and well tolerated. The probiotic intervention did not cause disruptive alterations in the composition or metabolic activity of health-associated microbiota. The results presented pave the way for the exploration of the impact of the strain on microbiota alterations in a clinical setting.

## Abstract (precis)

This study is the first to determine the safety and tolerance in humans of a butyrate-producing Clostridium cluster IV next-generation probiotic. Advances in gut microbiota research have triggered interest in developing colon butyrate producers as next-generation probiotics. Butyricicoccus pullicaecorum 25-3^T^ is one such potential probiotic, with demonstrated safety *in vitro* as well as in animal models. Here, we produced an encapsulated B. pullicaecorum formulation that largely preserved its viability over an 8-month storage period at 4°C. Administration of this formulation to healthy volunteers allowed us to establish the intervention as safe and well tolerated. The probiotic intervention did not cause disruptive alterations in the composition or metabolic activity of health-associated microbiota. The results presented pave the way for the exploration of the impact of the strain on microbiota alterations in a clinical setting.

## INTRODUCTION

Due to its close association with host health, the human gut microbiota is widely considered a promising target for preventive and therapeutic interventions (1–3). Based on the assumption of a causal or cocausal implication of microbiota alterations in the development or persistence of suboptimal health or disease conditions, several approaches to modulate the composition or metabolic activity of the gut microbial community have been proposed (4). Such microbiota modulation strategies aim at restoring ecosystem eubiosis by introducing or promoting growth of beneficial bacteria or bacterial consortia (5, 6). Ultimately, even the replacement of a dysbiotic bacterial community by a health-associated microbiota through fecal transplantation can be envisaged (7).

A long-standing microbiota modulation approach is the use of probiotics, live microorganisms that, when administered in adequate amounts, confer a health benefit on the host (6). For many years, probiotic research has mainly—although not exclusively (8, 9)—revolved around bifidobacteria and lactic acid bacteria (6). Lately, however, following up on new insight into the interactions between the gut microbiota and the human host, a whole new range of gut isolates have drawn the attention of the probiotic community (10). Such next-generation probiotics are rather broadly defined as probiotics that have not been used as agents to promote health to date (10). A particularly interesting category of such potential next-generation probiotics comprises Clostridium cluster IV/XIVa colon butyrate producers (11). The rationale underlying this interest is straightforward: butyrate is the major energy source for colonocytes, influences cell differentiation, and strengthens the epithelial defense barrier (12, 13). Notwithstanding some noteworthy exceptions (14), butyrate has repeatedly been shown to reduce intestinal inflammation (13), as reflected in the decreased abundance of butyrate producers in feces of inflammatory bowel disease (IBD) patients (15, 16). Hence, the administration of colon butyrate producers could become an essential part of IBD management by counteracting dysbiosis and promoting overall gut health (17).

Isolated from the cecum of broiler chickens (18), Butyricicoccus pullicaecorum 25-3^T^ is a Gram-positive, strictly anaerobic Clostridium cluster IV bacterium that produces high levels of butyrate (18). Following up on its observed reduced relative abundance in fecal samples of IBD patients (19), the safety and probiotic potential of the strain have been assessed throughout a series of *in vitro* and animal experiments. Whole-genome sequencing indicated B. pullicaecorum to be nonvirulent, with limited antibiotic resistance potential (20). B. pullicaecorum safety has been demonstrated in rats through both standard acute and 28-day repeated oral dose toxicity tests (20). The bacterium was shown to be intrinsically tolerant to stomach and small intestine conditions (21). Regarding its potential anti-inflammatory properties, B. pullicaecorum cell culture supernatant enhanced barrier integrity in inflamed CaCo-2 epithelial cells (19). Overall, B. pullicaecorum has gained the status of a promising exponent of the recent wave of next-generation probiotics that are currently making their way into clinical practice.

Here, in line with the recommendations of World Health Organization (22), we assessed the safety and tolerability of B. pullicaecorum in an exploratory phase 1 trial (ClinicalTrials.gov identifier NCT02477033). First, we up-scaled production of the strain and designed a protocol allowing stable encapsulation. Next, we performed what is to our knowledge the first randomized, double-blind, placebo-controlled crossover trial in healthy volunteers with a butyrate-producing Clostridium cluster IV bacterium. Evaluation endpoints comprised the impact of B. pullicaecorum administration on subjects’ health, fecal microbiome composition, and stool metabolome profiles. The present study represents a crucial step in the ongoing exploration of the probiotic potential of B. pullicaecorum.

## RESULTS

### A stable *Butyricicoccus pullicaecorum* formulation.

Given the often strict anaerobic metabolism of the bacterial strains of interest (23), production and conservation represent major challenges in the development of next-generation probiotic formulations suited for human consumption. Here, we cultured B. pullicaecorum 25-3^T^ under anaerobic conditions, and the lyophilized culture was used to fill hydroxypropyl methylcellulose (HPMC) capsules at a concentration of 10^8^ CFU/capsule—the maximal dose that fit in the capsules. Sealed and coated capsules remained intact after 2 h in 0.1 M HCl and disintegrated after 17 min at pH 6.8. Capsules were stored in aluminum sachets at 4°C. Eight months after production (4 months after completion of the study), bacterial viability was assessed as a measure for product stability. On average, capsules were found to contain 6.7 × 10^7^ CFU (67% viability), indicating an acceptable shelf life of the probiotic formulation.

### Administration of *Butyricicoccus pullicaecorum* is safe and well tolerated.

**FIG 1:**
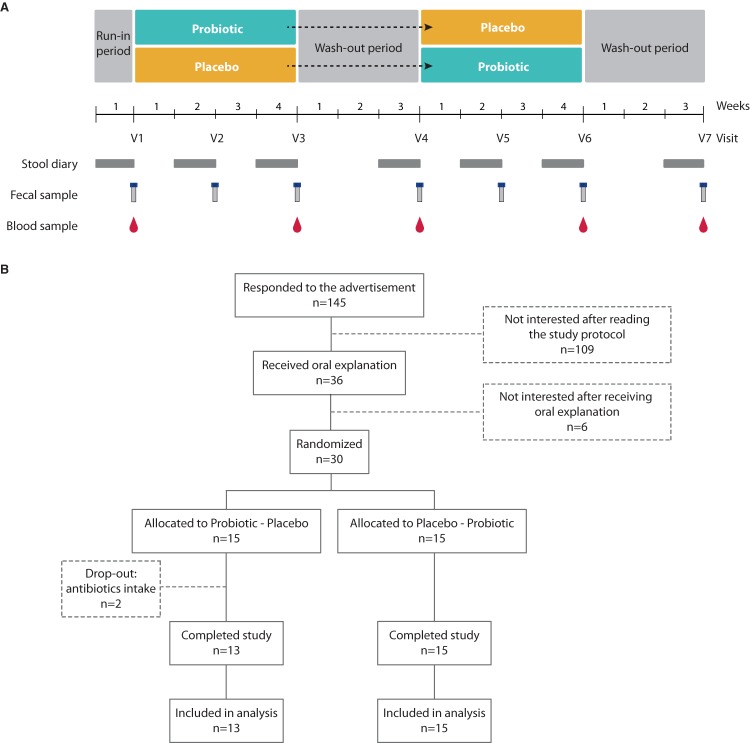
Study design. (A) Schematic representation of the study design. Stool diaries and samples were collected as depicted. V1 to V7 represent study visits before, during, and after each intervention and/or washout period. (B) Participant flow diagram showing the number of participants at each stage and reason for dropping out.

To evaluate safety of B. pullicaecorum administration, we set up a randomized, double-blind, placebo-controlled crossover trial with healthy volunteers. Thirty healthy subjects (16 female and 14 male; age, 22 to 52 years; body mass index [BMI], 18.9 to 27.8 kg/m^2^) were recruited and randomized over two intervention sequences between February and June 2014. Both groups were balanced according to gender, age, BMI, and smoking habits (Table 1). In addition, no differences in medication intake were detected (chi-square test [χ^2^] = 0.14 and *P* = 0.712, with intake of medication affecting intestinal transit or gut microbiota among the exclusion criteria [Table 1]), and participants were instructed to follow their usual diet throughout the study. The study setup covered a 1-week run-in and two 4-week intervention periods, each followed by a washout of 3 weeks (Fig. 1). Two subjects from one group dropped out of the study due to antibiotic treatment during the first intervention period and were excluded from further analyses. Compliance rates were similar between the treatment and placebo intervention periods (98% of capsules provided were effectively taken in both groups). Baseline values of the study’s primary outcome variables did not differ significantly from those observed after each washout period, implying that no carryover effects between both interventions were to be expected.

**TABLE 1:** Baseline characteristics of the study population according to randomization group

Characteristic	Result for:		*P* value
	Probiotic-placebo group	Placebo-probiotic group	
Sex, no. male/female	7/8	7/8	1.000
Age, yr (range)	32 (26–45)	28 (25–33)	0.176
BMI, kg/m²	23.6 ± 2.1	22.1 ± 1.9	0.064
Smoking status, no. yes/no/ex-smoker	1/13/1	1/10/4	0.314
Medication intake, no. yes/no^*a*^	7/8	6/9	0.712
Smoking pack years	0 (0–0)	0 (0–0.5)	0.288

a Intake of medication known to affect microbiota composition or gastrointestinal transit time (including antibiotics, prebiotics, and other probiotics) the preceding month or during the study was part of the exclusion criteria.

Daily administration of B. pullicaecorum capsules for 4 weeks was well tolerated by all participants. No severe adverse events (SAEs) were reported, and the numbers of reported adverse events (AEs) did not differ significantly between the treatment and placebo periods (Table 2). Participants maintained their normal bowel habits (stool frequency and consistency and occurrence of abdominal pain, bloating, or other abdominal pain) during the B. pullicaecorum intervention (Table 2). Changes in fecal calprotectin levels upon treatment did not differ from those observed over the placebo intervention, indicating that B. pullicaecorum administration did not elicit intestinal inflammation: the median was 1.1 µg/g (interquartile range [IQR], −4.3 to 14.4 µg/g) versus 8.5 µg/g (IQR, 6.7 to 46.5 µg/g) (*P* = 0.264). Finally, no alterations in variation of blood chemistry parameters encompassing hematology values, liver and kidney function, blood minerals, and lipids were observed when comparing placebo and treatment interventions (see Table S1 in the supplemental material). The primary endpoints of the study were thus successfully met.

**TABLE 2:** Occurrence of adverse events during the probiotic and placebo intervention period^*a*^

Symptom	Severity	No. of events occurring during administration of:		*P* value
		Probiotic (*n* = 28)	Placebo (*n* = 28)	
Abdominal pain	Mild	1	1	0.157
	Moderate	0	1	
Allergic reaction	Mild	0	1	1.000
Arthralgia	Mild	1	0	1.000
Diarrhea	Mild	1	3	0.577
	Moderate	1	1	
Gastritis and enterocolitis	Moderate	1	1	1.000
GI disorders—other, loose stools	Mild	4	5	1.000
Laryngeal inflammation	Moderate	0	1	1.000
Myositis	Mild	0	1	1.000
Toothache	Mild	0	1	1.000

a Values were compared with McNemar tests when only one grade of severity was reported (mild or moderate) and with Wilcoxon signed-rank tests when two grades were reported. GI, gastrointestinal; *n*, number of subjects.

10.1128/mSystems.00094-18.1 TABLE S1 Probiotic and placebo effects on bowel habits and blood chemistry parameters (hematology values, liver and kidney functioning, blood minerals, and lipids) measured after 4 weeks of intake. Download Table S1, PDF file, 0.1 MB. Copyright © 2018 Boesmans et al. 2018 Boesmans et al. This content is distributed under the terms of the Creative Commons Attribution 4.0 International license.

### *Butyricicoccus pullicaecorum* administration does not disrupt microbial community structure.

**FIG 2:**
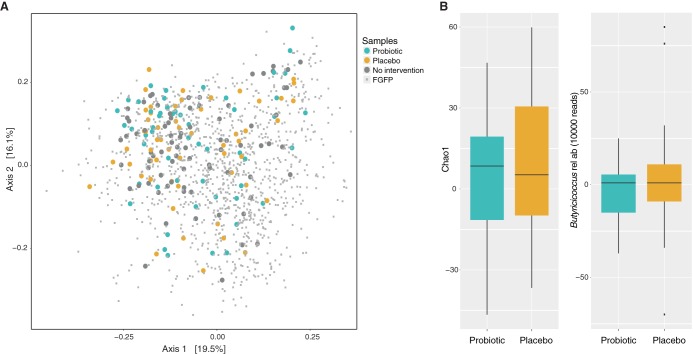
(A) Principal-coordinate analysis (PCoA) of interindividual differences in microbiota composition (Bray-Curtis dissimilarity) with samples included in the analysis (*n* = 188) and 1,106 samples from the Flemish Gut Flora Project. Samples collected during the intervention trial fell within ranges of normal variation, and no separation was observed after intervention compared to baseline. (B) Variation in microbiota richness (Chao1) and in relative abundances of the genus Butyricicoccus after probiotic administration compared to placebo. No significant differences were observed (*r* = −0.05 and *P* = 0.747 and *r* = −0.12 and *P* = 0.363, respectively).

Next, we assessed the potential impact of B. pullicaecorum administration on the health-associated microbiota community structure as reflected in fecal material from healthy volunteers. The microbiome composition of 188 out of 196 fecal samples collected during the intervention trial fell within the ranges of normal variation as observed within the Flemish Gut Flora Project data set (24) (8 samples had a read count of <10,000 and were excluded from analyses) (Fig. 2A). To quantify the effect of B. pullicaecorum supplementation on community structure, we compared microbiome dissimilarities (beta-diversity, expressed as Bray-Curtis dissimilarity index) between the start and the end of each intervention period. We could not observe any difference between the impact of probiotic treatment and placebo (Wilcoxon signed-rank test, *r* = −0.01, *P* = 0.94). Likewise, compared to placebo, the probiotic did not affect community stability, as reflected by microbiome richness (Chao1, *r* = −0.05, *P* = 0.747 [Fig. 2B]) or evenness (Pielou, *r* = −0.16, *P* = 0.250). B. pullicaecorum administration did not cause any significant changes in abundances of single genera (see Table S2 in the supplemental material). Of note, no accumulation of the treatment genus over the intervention study was observed (*r* = −0.12, *P* = 0.363 [Fig. 2B]), indicating transient colonization of the ecosystem. Overall, we can state that the B. pullicaecorum formulation administered was well tolerated both by the healthy human participants and by their health-associated intestinal microbiota.

10.1128/mSystems.00094-18.2 TABLE S2 Differences in relative abundances of microbial genera after probiotic administration (end − start) compared to placebo (end − start). Download Table S2, PDF file, 0.1 MB. Copyright © 2018 Boesmans et al. 2018 Boesmans et al. This content is distributed under the terms of the Creative Commons Attribution 4.0 International license.

### Fecal metabolite profiles remain stable throughout *Butyricicoccus pullicaecorum* intervention.

**FIG 3:**
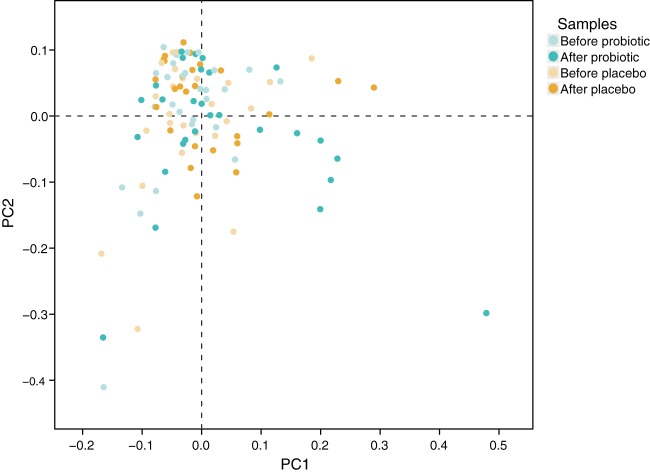
Fecal metabolite profile analyzed by PLS-DA. No significant difference is found between metabolomes after probiotic intervention compared to baseline (*P* > 0.05 by RDA).

As compositional microbiome stability does not necessarily exclude fluctuations in microbiota metabolic activity, we assessed the impact of the B. pullicaecorum intervention on fecal metabolite profiles. In total, we relatively quantified 314 volatile organic compounds (VOCs) in 140 samples (study visits V1, V3, V4, V6, and V7) from 28 study volunteers, with an average of 88 VOCs per sample. Nineteen VOCs were detected in all samples, 55 occurred in >80% of the fecal aliquots analyzed, and 64 were characterized as sample specific. Changes induced in the number of VOCs detected per sample by treatment did not differ from those observed upon placebo intervention (1.2 ± 9.5 versus −3.1 ± 8.5; *P* = 0.101). Metabolite profiles of samples collected before and after B. pullicaecorum or placebo intervention could not be discriminated (partial least-squares discriminant analysis [PLS-DA]) (Fig. 3). Accordingly, probiotic treatment did not lead to significant shifts in metabolite relative concentrations compared to placebo and baseline samples (redundancy analysis [RDA], *P* = 0.468).

To investigate the potential impact of B. pullicaecorum administration on gut saccharolytic and proteolytic fermentation processes, absolute quantification of a number of selected marker metabolites was performed. The short-chain fatty acids (SCFAs) acetate, propionate, and butyrate were included as indicators of saccharolytic fermentation, while dimethyl sulfide, *p*-cresol, indole, and the branched-chain fatty acids (BCFAs) isobutyrate and isovalerate reflect proteolytic metabolism (25). Probiotic-induced variation in selected marker metabolite concentrations did not differ significantly from fluctuations observed over placebo intervention (Table 3). Of note, also fecal butyrate was not differentially affected by B. pullicaecorum and placebo intervention (two-sample *t* test, *P* = 0.613). Overall, we can conclude that B. pullicaecorum consumption did not significantly alter microbial metabolite profiles as observed in fecal material from healthy individuals.

**TABLE 3:** Probiotic and placebo effects on absolutely quantified colonic fermentation metabolites after 4 weeks of intake

Metabolite	Chemical class^*a*^	Metabolite concentration with^*b*^:				*P* value for probiotic vs placebo
		Probiotic		Placebo		
		Start	End	Start	End	
Acetate (mM)	SCFA	162.14 ± 75.53	176.14 ± 81.55	181.68 ± 80.74	192.28 ± 85.31	0.869
Propionate (mM)	SCFA	42.49 ± 27.53	38.86 ± 17.21	40.33 ± 21.29	41.20 ± 17.85	0.436
Butyrate (mM)	SCFA	28.31 ± 15.29	29.90 ± 15.42	30.12 ± 15.85	34.09 ± 19.13	0.613
Isobutyrate (mM)	BCFA	3.15 ± 1.29	3.57 ± 1.71	3.15 ± 1.35	3.46 ± 1.57	0.815
Isovalerate (mM)	BCFA	1.94 ± 0.79	2.37 ± 1.19	2.07 ± 0.96	2.26 ± 1.20	0.457
Dimethyl sulfide (µM)	S-compound	0.01 (0.01–0.01)	0.01 (0.01–0.01)	0.01 (0.01–0.01)	0.01 (0.01–0.01)	0.855
*p*-Cresol (mM)	Phenol	0.61 ± 0.39	0.73 ± 0.49	0.68 ± 0.56	0.69 ± 0.58	0.498
Indole (µM)	Indole	2.79E−3 (1.20E−4 to 8.76E−3)	1.93E−3 (0 to 8.02E−3)	1.98E−3 (9.32E−4 to 4.61E−3)	3.09E−3 (1.38E−3 to 6.43E−3)	0.81

a SCFA, short-chain fatty acid; BCFA, branched-chain fatty acid.

b Except as noted, values are expressed as mean ± SD and were compared with paired two-sample *t* tests when the normality assumption was met. If not, data are represented as median with IQR in parentheses and were compared with Wilcoxon signed-rank tests.

## DISCUSSION

Advances in gut microbiota research have revived interests in developing novel probiotic applications. While preclinical evidence *in vitro* or in animal models has been shown for several of such next-generation probiotics, few have been tested in humans (10). These include Clostridium butyricum MIYAIRI 588 (Clostridium cluster I), demonstrated as safe *in vitro* and in rodents (26) and moderately effective in treating Helicobacter pylori infections (27), antibiotic-associated diarrhea (28), and preventing formation of postsurgery pouchitis in ulcerative colitis patients (29), and Bacteroides xylanisolvens DSM 23694, which induced generation of antibodies against the cancer-specific antigen TFα (30). Given the reported anti-inflammatory properties of butyrate (13) and the decreased abundances of butyrate-producing bacteria observed in IBD patients (15, 16), colon butyrate producers are particularly promising as niche-specific next-generation probiotics. Administration of probiotic colon butyrate producers could potentially exert beneficial effects in patients, reducing intestinal inflammation and restoring eubiosis. B. pullicaecorum 25-3^T^ has been demonstrated to be safe in *in vitro* as well as animal models (20). The strain is intrinsically tolerant to the harsh conditions of the stomach (low pH) and the small intestine (presence of bile salts and pancreatic enzymes), indicating the potential to reach the colon in a viable and metabolically active state (21). In addition, the supernatant of the cultured strain also reduced inflammation and prevented epithelial integrity loss in human cell lines (31). However, production and conservation of strictly anaerobic probiotic bacteria for human consumption remain challenging. Here, we first cultured B. pullicaecorum 25-3^T^, followed by encapsulation (10^8^ CFU) and pH-resistant coating. Eight months after production (4 months after completion of the study), viability remained at 67%, indicating stability and an acceptable shelf life of the probiotic formulation (32).

Next, we performed a randomized, double-blind, placebo-controlled crossover trial with 30 healthy volunteers. Very high compliance rates were attained (98% of the capsules provided were effectively taken), and no carryover effects were detected. The primary endpoints of the study were achieved, with daily administration of B. pullicaecorum being safe and well tolerated, as determined by the absence of differences between the probiotic and placebo interventions in the occurrence of (severe) adverse events, blood chemistry parameters, changes in bowel habits, and intestinal inflammation markers. While we would envisage reduced intestinal inflammation in patients with gastrointestinal inflammation, no changes were expected in the present safety trial with healthy individuals.

Secondary endpoints of the study included effects of the probiotic formulation on microbiota composition and metabolic activity. B. pullicaecorum administration did not disrupt microbial community structure, and no alterations in relative abundances of specific microbial taxa were detected. Furthermore, there was no accumulation of Butyricicoccus sequences over the intervention, and thus, we can conclude that the probiotic did not persist in the colon of the study participants. The microbial metabolic activity also remained stable throughout the intervention. No significant increase in fecal butyrate levels measured was detected after probiotic compared to placebo intervention. While we cannot rule out this being a consequence of the probiotic dosage used in the study, fecal measurements have been challenged as a readout of colonic microbial butyrate production. As up to 95% of SCFAs are estimated to be rapidly absorbed by colonocytes, the fraction excreted in feces only reflects the ratio between production and absorption rates and not the *in situ* production of the metabolites (26).

In conclusion, this randomized placebo-controlled crossover study demonstrated safety of B. pullicaecorum 25-3^T^ administration to healthy subjects. The strain is not only well tolerated by the human host, but also does not cause any disruptive changes in the composition or metabolic activity of a health-associated gut microbiota. Hence, as a further step in the development of B. pullicaecorum as a next-generation probiotic, an intervention study using a therapeutic dosage of the strain grown in an adjusted, food-grade culture medium in a clinical setting can be envisaged to study the strain’s effect on disease-associated microbiota alterations and the accompanying impact on host health and well-being.

## MATERIALS AND METHODS

### Study design. (i) Study population.

Thirty healthy subjects were recruited among students of the KU Leuven and employees of the University Hospital of Leuven. All subjects were generally healthy, had a regular eating pattern, were free of medication affecting intestinal transit or gut microbiota, and did not take any pre-, pro-, or antibiotics during the month preceding the study. None of the subjects had a history of gastrointestinal (GI) disease (IBD, IBS [irritable bowel syndrome], or diarrhea) or abdominal surgery (with the exception of appendectomy). Additional exclusion criteria were following a weight loss diet during the month preceding the study, maintaining strict dietary habits (e.g., veganism), pregnancy or breastfeeding, and intake of more than 10 alcoholic drinks per week. Subjects were instructed to maintain their usual diet during the study period and to avoid any intake of pre- and other probiotics. The study was approved by the Ethics Committee of the University Hospitals Leuven (ML9449). All subjects gave their written informed consent prior to enrollment. The trial was registered at ClinicalTrials.gov (NCT02477033).

### (ii) Study product.

The bacterial strain Butyricicoccus pullicaecorum 25-3^T^ (LMG 24109^T^; CCUG 55265^T^) was cultured in M2GSC broth at pH 6 for 24 h at 37°C under anaerobic conditions (84% N_2_, 8% CO_2_, 8% H_2_) as described by Miyazaki et al. (27), with the addition of 15% (vol/vol) clarified rumen fluid instead of 30%. After overnight incubation, bacteria were collected by centrifugation (10 min, 5,000 × *g*, 37°C), resuspended in a lyoprotectant (consisting of horse serum supplemented with 7.5% trehalose and 1 mg/ml cysteine-HCl, pH 6). All manipulations were performed under anaerobic conditions (84% N_2_, 8% CO_2_, 8% H_2_). The suspensions were freeze-dried overnight using an Alpha 1-2 LDplus (Christ, Osterode, Germany) freeze drier under default operation conditions. Cultivability of the strain was determined through anaerobic plating of serial dilutions on M2GSC agar (16). Hydroxypropyl methylcellulose (HPMC) size 0 capsules were manually filled with 400 mg lyophilized product at a concentration of 10^8^ CFU/capsule. The capsules were sealed and coated with a pH-resistant coating consisting of the enteric polymer cellulose acetate phthalate (CAP) and the plasticizer diethyl phthalate by SEPS Pharma NV (Ghent, Belgium). Placebo capsules were filled with maltodextrin (Paselli MD 6; Avebe, Veendam, The Netherlands) and coated as described above. All capsules were stored in heat-sealed aluminum sachets at 4°C. Eight months after capsule production, bacterial viability was determined as a measure for product stability.

### (iii) Study setup.

The study was set up as a randomized, double-blind, placebo-controlled crossover trial, conducted between February and June 2014. Study design included a 1-week run-in period and two interventions of 4 weeks, each followed by a washout of 3 weeks (Fig. 1). Volunteers were randomly allocated to one of the randomization groups at a 1:1 ratio, starting with either the probiotic or placebo intervention period. Block randomization of the subjects was performed by an independent researcher who was not involved in the study using an online randomization tool (www.randomization.com) with a fixed block size of four, stratified for sex and visit sequence. Probiotic and placebo capsules were sealed in identical containers and appointed to the subjects by the independent researcher. Subjects as well as the study researchers were blind to the intervention sequence until termination of all analytical assessments. During the run-in week, study volunteers were asked to fill in a defecation journal and GI questionnaire. After the run-in period, participants visited the lab to provide a fasted blood sample and to deposit a fecal sample collected in the 24 h preceding the visit and stored intermediately at 4°C. During the first intervention period, subjects consumed daily one capsule containing the bacterial strain (10^8^ CFU [treatment intervention]) or maltodextrin (placebo intervention) at breakfast for 4 weeks. After a washout period of 3 weeks, subjects switched to the alternative intervention again for 4 weeks, followed by a final 3-week washout. Fecal and blood samples were collected after weeks 2 and 4 of the intervention periods and at the end of each washout period. During the week preceding sampling, participants kept a defecation journal and completed a GI questionnaire. At each lab visit, subjects were asked to report changes in medication and the occurrence of adverse events during the preceding period. Adverse events were categorized and graded on their severity according to the Common Terminology Criteria for Adverse Events version 4 (28). Participants were instructed to return the remaining capsules after each intervention period to check compliance.

### (iv) Study endpoints.

The primary endpoint of the trial was the assessment of safety and tolerability of B. pullicaecorum administration in healthy subjects. To do so, GI complaints, stool parameters, blood parameters, and fecal calprotectin concentrations were determined. As secondary outcome variables, the impact of the bacterial strain on microbial composition and activity was assessed.

### Analytical methods. (i) Defecation journals.

Defecation journals contained daily information on stool frequency, stool consistency (Bristol stool score), and GI symptoms, such as abdominal pain and bloating. Parameters were averaged per week to obtain one value per parameter for each study visit. Additionally, participants reported symptom scores at the end of each week based on overall abdominal pain, bloating, defecation and stool specifications, and abdominal complaints during that week.

### (ii) Blood parameters.

Hematological parameters, liver and kidney function parameters, and blood lipids and minerals were quantified using standard laboratory techniques.

### (iii) Fecal calprotectin.

To measure fecal calprotectin, a marker of intestinal inflammation, stool aliquots were extracted using the Smart-Prep fecal sample preparation kit (Bühlmann Laboratories AG, Schönenbuch, Switzerland), and extracts were kept at *−*20°C. Afterwards, fecal calprotectin was quantified using a sandwich immunoassay (Quantum Blue quantitative calprotectin lateral flow assay; Bühlmann Laboratories AG) following the manufacturer’s instructions. Calprotectin concentrations were expressed in µg/g stool.

### (iv) Gut microbiota composition.

Microbial DNA was extracted from frozen fecal samples using the PowerMicrobiome RNA isolation kit (Mo Bio Laboratories, Inc., Carlsbad, CA) as described previously (24). 16S rRNA genes were amplified using the 515F/806R primer set, targeting the V4 hypervariable region (29). Sequencing was performed using the Illumina MiSeq platform with sequencing kit MiSeq v2, producing 250-bp paired-end reads. For sequence analysis, fastq sequences were merged using FLASH version 1.2.10 (30) and quality filtered (threshold: >90% of nucleotides should have a quality score of ≥25) with the FASTX-Toolkit v0.0.14 (http://hannonlab.cshl.edu/fastx_toolkit/). Chimera removal was performed using the UCHIME algorithm in USEARCH v6.0.307 (31), and taxonomical assignment of sequences was performed with the RDP classifier v2.12 (32). Phylum-to-genus matrices were subsequently created using Perl scripts. Samples were rarefied to 10,000 randomly selected reads, with samples with <10,000 reads (*n* = 8) excluded from the analysis.

### (v) Fecal metabolite profiles.

Fecal aliquots of 125 mg were suspended in a total volume of 5 ml H_2_O, together with a pinch of Na_2_SO_4_ (99%; Acros, Geel, Belgium) to salt out the solution, 130 µl of H_2_SO_4_ (98%; Merck, Darmstadt, Germany) for acidification, a magnetic stirrer, and 20 µl of internal standard (2*-*ethyl butyrate [1.6 mg/liter, 99%; Merck], diethyl sulfide [0.047 mg/liter, 98%; Sigma-Aldrich, Steinheim, Germany], and 2,6-dimethyl phenol [0.162 mg/liter, 99.5%; Sigma-Aldrich]). Volatile organic compounds (VOCs) were analyzed using a gas chromatography-mass spectrometry (GC-MS) quadrupole system (Trace GC Ultra and DSQ II; Thermo Electron Corporation, Waltham, MA), coupled on-line to a purge-and-trap system (Velocity; Teldyne Tekmar, Mason, OH), as previously described (33). The VOCs were separated on an analytical column (AT Aquawax DA, 30 m by 0.25-mm inside diameter [i.d.], 0.25-μm film thickness; Grace, Deerfield, IL), and masses were detected between *m/z* 33 and 200 at 1.5 full scans/s. The resulting chromatograms were processed using AMDIS (Automatic Mass Spectral Deconvolution and Identification Software version 2.71) provided by the National Institute of Standards and Technology (NIST). This software uses adjacent peak deconvolution and background subtraction to acquire clarified spectra from the overlapping peaks. The mass spectra of unknown peaks were then compared to the NIST library and were positively identified when having a match factor of ≥90%. Relative indices (RIs) of all VOCs were calculated versus 2-ethyl butyrate as an internal standard, and VOCs were classified according to chemical class. The resulting metabolite profiles were organized in a three-dimensional data matrix using sample names (observations), identified metabolites (variables), and normalized peak intensity versus 2-ethyl butyrate (variable indices). A number of metabolites were selected as markers for saccharolytic fermentation (the short-chain fatty acids [SCFAs] acetic acid, propionic acid, and butyric acid) and proteolytic fermentation (dimethyl sulfide, *p*-cresol, indole, and the branched-chain fatty acids [BCFAs] isobutyric acid and isovaleric acid). They were absolutely quantified using appropriate calibration curves obtained with internal standard quantification. SCFAs and BCFAs were quantified using 2-ethyl butyrate as the corresponding internal standard, whereas *p*-cresol and indole were quantified versus 2,6-dimethyl phenol and dimethyl sulfide versus diethyl sulfide.

### Statistical analysis. (i) Study population characteristics.

Differences between the visit after an intervention period and that preceding this period were calculated to compare effects of the interventions (probiotic effect and placebo effect). Assumptions of normality were explored using the Shapiro-Wilk test. When the assumption was met, differences were evaluated using paired two-sample *t* tests, and values were expressed as mean ± standard deviation (SD). When the normality assumption was not met or when the data were ordinal, Wilcoxon signed-rank tests were performed and data were presented as median with interquartile range (IQR) in parentheses. Similarly, when data comparisons were unpaired, unpaired two-sample *t* tests and Mann-Whitney U tests were used, respectively. Binary data were compared using the Pearson chi-square test when unpaired and with the McNemar test in the case of paired data. For nominal data, the likelihood-ratio chi-square test was applied. Results were corrected for multiple testing using the Benjamini and Hochberg false-discovery rate (FDR) correction (27). Statistical analyses were performed using the R statistical software (34). The level of statistical significance was set at *P* < 0.05, with *P* < 0.1 considered a trend toward significance.

### (ii) Microbiota composition.

Statistical analysis of microbiota composition and graphical representations were performed in R (version 3.4.3), using the packages vegan (35), phyloseq (36), coin (37), and ggplot2 (38). Beta-diversity (Bray-Curtis dissimilarity index) and alpha-diversity, including richness (observed) and evenness (Pielou’s index), were calculated using the vegan R package on genus-level relative abundance matrices. Intervention-associated variations in relative abundances of microbial taxa were assessed in genera present in at least 15% of the samples and with a mean relative abundance of ≥1e−4. The influence of probiotic intervention on microbial diversity indices and taxa compared to placebo (difference between samples before and after intervention) was assessed with Wilcoxon signed-rank tests. Correction for multiple testing (Benjamini-Hochberg procedure, FDR) was applied, and significance was defined at an FDR of <10%.

### (iii) Metabolite profiles.

Sample-specific metabolites were removed from the analysis as they do not exert any discriminatory power (39). Principal-component analysis (PCA) was applied to detect outliers (*n* = 3), which were excluded from further analysis. Partial least-squares discriminant analysis (PLS-DA) with full cross-validation was performed with the mdatools R package (40) to cluster samples with similar metabolite profiles according to the intervention, and the result was presented as a score plot. Corresponding loading plots showing the metabolites were used to identify components accounting for that discrimination. The influence of probiotic treatment on metabolite profiles was determined by redundancy analysis (RDA) using the vegan R package (35).

### Data availability.

Data have been made available at the European Nucleotide Archive under accession no. PRJEB29261.

## References

[1] Lloyd-Price J, Abu-Ali G, Huttenhower C 2016 The healthy human microbiome. Genome Med 8:51. doi:10.1186/s13073-016-0307-y. 27122046 PMC4848870

[2] Lynch SV, Pedersen O 2016 The human intestinal microbiome in health and disease. N Engl J Med 375:2369–2379. doi:10.1056/NEJMra1600266. 27974040

[3] Cho I, Blaser MJ 2012 The human microbiome: at the interface of health and disease. Nat Rev Genet 13:260–270. doi:10.1038/nrg3182. 22411464 PMC3418802

[4] Walsh CJ, Guinane CM, O'Toole PW, Cotter PD 2014 Beneficial modulation of the gut microbiota. FEBS Lett 588:4120–4130. doi:10.1016/j.febslet.2014.03.035. 24681100

[5] Gibson GR, Hutkins R, Sanders ME, Prescott SL, Reimer RA, Salminen SJ, Scott K, Stanton C, Swanson KS, Cani PD, Verbeke K, Reid G 2017 Expert consensus document: the International Scientific Association for Probiotics and Prebiotics (ISAPP) consensus statement on the definition and scope of prebiotics. Nat Rev Gastroenterol Hepatol 14:491–502. doi:10.1038/nrgastro.2017.75. 28611480

[6] Hill C, Guarner F, Reid G, Gibson GR, Merenstein DJ, Pot B, Morelli L, Canani RB, Flint HJ, Salminen S, Calder PC, Sanders ME 2014 Expert consensus document. The International Scientific Association for Probiotics and Prebiotics consensus statement on the scope and appropriate use of the term probiotic. Nat Rev Gastroenterol Hepatol 11:506–514. doi:10.1038/nrgastro.2014.66. 24912386

[7] Cammarota G, Ianiro G, Tilg H, Rajilić-Stojanović M, Kump P, Satokari R, Sokol H, Arkkila P, Pintus C, Hart A, Segal J, Aloi M, Masucci L, Molinaro A, Scaldaferri F, Gasbarrini G, Lopez-Sanroman A, Link A, de Groot P, de Vos WM, Högenauer C, Malfertheiner P, Mattila E, Milosavljević T, Nieuwdorp M, Sanguinetti M, Simren M, Gasbarrini A, European FMT Working Group. 2017 European Consensus Conference on faecal microbiota transplantation in clinical practice. Gut 66:569–580. doi:10.1136/gutjnl-2016-313017. 28087657 PMC5529972

[8] Hager CL, Ghannoum MA 2017 The mycobiome: role in health and disease, and as a potential probiotic target in gastrointestinal disease. Dig Liver Dis 49:1171–1176. doi:10.1016/j.dld.2017.08.025. 28988727

[9] Sonnenborn U, Schulze J 2009 The non-pathogenic Escherichia coli strain Nissle 1917—features of a versatile probiotic. Microb Ecol Health Dis 21:122–158. doi:10.3109/08910600903444267.

[10] O'Toole PW, Marchesi JR, Hill C 2017 Next-generation probiotics: the spectrum from probiotics to live biotherapeutics. Nat Microbiol 2:17057. doi:10.1038/nmicrobiol.2017.57. 28440276

[11] Van Immerseel F, Ducatelle R, De Vos M, Boon N, Van De Wiele T, Verbeke K, Rutgeerts P, Sas B, Louis P, Flint HJ 2010 Butyric acid-producing anaerobic bacteria as a novel probiotic treatment approach for inflammatory bowel disease. J Med Microbiol 59:141–143. doi:10.1099/jmm.0.017541-0. 19942690

[12] Hamer HM, Jonkers D, Venema K, Vanhoutvin S, Troost FJ, Brummer R-J 2008 The role of butyrate on colonic function. Aliment Pharmacol Ther 27:104–119. doi:10.1111/j.1365-2036.2007.03562.x. 17973645

[13] Louis P, Hold GL, Flint HJ 2014 The gut microbiota, bacterial metabolites and colorectal cancer. Nat Rev Microbiol 12:661–672. doi:10.1038/nrmicro3344. 25198138

[14] Lakhdari O, Tap J, Béguet-Crespel F, Le Roux K, de Wouters T, Cultrone A, Nepelska M, Lefèvre F, Doré J, Blottière HM 2011 Identification of NF-κB modulation capabilities within human intestinal commensal bacteria. J Biomed Biotechnol 2011:1. doi:10.1155/2011/282356. PMC3134244 21765633

[15] Lepage P, Häsler R, Spehlmann ME, Rehman A, Zvirbliene A, Begun A, Ott S, Kupcinskas L, Doré J, Raedler A, Schreiber S 2011 Twin study indicates loss of interaction between microbiota and mucosa of patients with ulcerative colitis. Gastroenterology 141:227–236. doi:10.1053/j.gastro.2011.04.011. 21621540

[16] Frank DN, Robertson CE, Hamm CM, Kpadeh Z, Zhang T, Chen H, Zhu W, Sartor RB, Boedeker EC, Harpaz N, Pace NR, Li E 2011 Disease phenotype and genotype are associated with shifts in intestinal-associated microbiota in inflammatory bowel diseases. Inflamm Bowel Dis 17:179–184. doi:10.1002/ibd.21339. 20839241 PMC3834564

[17] Sokol H, Pigneur B, Watterlot L, Lakhdari O, Bermúdez-Humarán LG, Gratadoux J-J, Blugeon S, Bridonneau C, Furet J-P, Corthier G, Grangette C, Vasquez N, Pochart P, Trugnan G, Thomas G, Blottière HM, Doré J, Marteau P, Seksik P, Langella P 2008 Faecalibacterium prausnitzii is an anti-inflammatory commensal bacterium identified by gut microbiota analysis of Crohn disease patients. Proc Natl Acad Sci U S A 105:16731–16736. doi:10.1073/pnas.0804812105. 18936492 PMC2575488

[18] Eeckhaut V, Van Immerseel F, Teirlynck E, Pasmans F, Fievez V, Snauwaert C, Haesebrouck F, Ducatelle R, Louis P, Vandamme P 2008 Butyricicoccus pullicaecorum gen. nov., sp. nov., an anaerobic, butyrate-producing bacterium isolated from the caecal content of a broiler chicken. Int J Syst Evol Microbiol 58:2799–2802. doi:10.1099/ijs.0.65730-0. 19060061

[19] Eeckhaut V, Machiels K, Perrier C, Romero C, Maes S, Flahou B, Steppe M, Haesebrouck F, Sas B, Ducatelle R, Vermeire S, Van Immerseel F 2013 Butyricicoccus pullicaecorum in inflammatory bowel disease. Gut 62:1745–1752. doi:10.1136/gutjnl-2012-303611. 23263527

[20] Steppe M, Van Nieuwerburgh F, Vercauteren G, Boyen F, Eeckhaut V, Deforce D, Haesebrouck F, Ducatelle R, Van Immerseel F 2014 Safety assessment of the butyrate-producing Butyricicoccus pullicaecorum strain 25-3(T), a potential probiotic for patients with inflammatory bowel disease, based on oral toxicity tests and whole genome sequencing. Food Chem Toxicol 72:129–137. doi:10.1016/j.fct.2014.06.024. 25007784

[21] Geirnaert A, Steyaert A, Eeckhaut V, Debruyne B, Arends JBA, Van Immerseel F, Boon N, Van de Wiele T 2014 Butyricicoccus pullicaecorum, a butyrate producer with probiotic potential, is intrinsically tolerant to stomach and small intestine conditions. Anaerobe 30:70–74. doi:10.1016/j.anaerobe.2014.08.010. 25179909

[22] FAO/WHO. 2002 Guidelines for the evaluation of probiotics in food. World Health Organization, Geneva, Switzerland.

[23] Marteau P 2013 Butyrate-producing bacteria as pharmabiotics for inflammatory bowel disease. Gut 62:1673. doi:10.1136/gutjnl-2012-304240. 23461897

[24] Falony G, Joossens M, Vieira-Silva S, Wang J, Darzi Y, Faust K, Kurilshikov A, Bonder MJ, Valles-Colomer M, Vandeputte D, Tito RY, Chaffron S, Rymenans L, Verspecht C, De Sutter L, Lima-Mendez G, D'hoe K, Jonckheere K, Homola D, Garcia R, Tigchelaar EF, Eeckhaudt L, Fu J, Henckaerts L, Zhernakova A, Wijmenga C, Raes J 2016 Population-level analysis of gut microbiome variation. Science 352:560–564. doi:10.1126/science.aad3503. 27126039

[25] Macfarlane GT, Gibson GR, Cummings JH 1992 Comparison of fermentation reactions in different regions of the human colon. J Appl Bacteriol 72:57–64. 1541601 10.1111/j.1365-2672.1992.tb04882.x

[26] Verbeke KA, Boobis AR, Chiodini A, Edwards CA, Franck A, Kleerebezem M, Nauta A, Raes J, van Tol EAF, Tuohy KM 2015 Towards microbial fermentation metabolites as markers for health benefits of prebiotics. Nutr Res Rev 28:42–66. doi:10.1017/S0954422415000037. 26156216 PMC4501371

[27] Miyazaki K, Martin J, Marinsek-Logar R, Flint H 1997 Degradation and utilization of xylans by the rumen anaerobe Prevotella bryantii (formerly P. ruminicola subsp. brevis) B14. Anaerobe 3:373–381. doi:10.1006/anae.1997.0125. 16887612

[28] National Cancer Institute. 2018 Common terminology criteria for adverse events (CTCAE) v4.0. Division of Cancer Treatment and Diagnosis, National Cancer Institute, Bethesda, MD.

[29] Caporaso JG, Lauber CL, Walters WA, Berg-Lyons D, Huntley J, Fierer N, Owens SM, Betley J, Fraser L, Bauer M, Gormley N, Gilbert JA, Smith G, Knight R 2012 Ultra-high-throughput microbial community analysis on the Illumina HiSeq and MiSeq platforms. ISME J 6:1621–1624. doi:10.1038/ismej.2012.8. 22402401 PMC3400413

[30] Magoč T, Salzberg SL 2011 FLASH: fast length adjustment of short reads to improve genome assemblies. Bioinformatics 27:2957–2963. doi:10.1093/bioinformatics/btr507. 21903629 PMC3198573

[31] Edgar RC, Haas BJ, Clemente JC, Quince C, Knight R 2011 UCHIME improves sensitivity and speed of chimera detection. Bioinformatics 27:2194–2200. doi:10.1093/bioinformatics/btr381. 21700674 PMC3150044

[32] Wang Q, Garrity GM, Tiedje JM, Cole JR 2007 Naive Bayesian classifier for rapid assignment of rRNA sequences into the new bacterial taxonomy. Appl Environ Microbiol 73:5261–5267. doi:10.1128/AEM.00062-07. 17586664 PMC1950982

[33] De Preter V, Van Staeyen G, Esser D, Rutgeerts P, Verbeke K 2009 Development of a screening method to determine the pattern of fermentation metabolites in faecal samples using on-line purge-and-trap gas chromatographic-mass spectrometric analysis. J Chromatogr A 1216:1476–1483. doi:10.1016/j.chroma.2008.12.095. 19167006

[34] R Core Team. 2015 R: a language and environment for statistical computing. R Foundation for Statistical Computing, Vienna, Austria https://www.R-project.org/.

[35] Oksanen J, Blanchet FG, Kindt R, Legendre P, Minchin PR, O’Hara RB, Simpson GL, Solymos P, Stevens MHH, Wagner H 2015 vegan: Community Ecology Package. R package version 2.2-1 R Foundation for Statistical Computing, Vienna, Austria https://cran.r-project.org/web/packages/vegan/index.html.

[36] McMurdie PJ, Holmes S 2013 phyloseq: an R package for reproducible interactive analysis and graphics of microbiome census data. PLoS One 8:e61217. doi:10.1371/journal.pone.0061217. 23630581 PMC3632530

[37] Hothorn T, Hornik K, van de Wiel MA, Zeileis A 2008 Implementing a class of permutation tests: the coin package. J Stat Softw 28:1–23. doi:10.18637/jss.v028.i08.27774042

[38] Wickham H 2009 ggplot2: elegant graphics for data analysis. Springer-Verlag, Berlin, Germany.

[39] De Preter V, Ghebretinsae AH, Abrahantes JC, Windey K, Rutgeerts P, Verbeke K 2011 Impact of the synbiotic combination of Lactobacillus casei Shirota and oligofructose-enriched inulin on the fecal volatile metabolite profile in healthy subjects. Mol Nutr Food Res 55:714–722. doi:10.1002/mnfr.201000442. 21280206

[40] Kucheryavskiy S 2017 mdatools: multivariate data analysis for chemometrics. R Package version 082. R Foundation for Statistical Computing, Vienna, Austria https://CRANR-project.org/package=mdatools.

