# Supplementary material for: Butyrate Producers as Potential Next-Generation Probiotics: Safety Assessment of the Administration of Butyricicoccus pullicaecorum to Healthy Volunteers
Source: mSystems. 2018 Nov 6;3(6):e00094-18. doi: 10.1128/mSystems.00094-18 (PMC6222043; doi:10.1128/mSystems.00094-18)
Supplement: TABLE S1 [file sys006182288st1.pdf]

Supplemental Table S1: Probiotic and placebo effects on bowel habits and blood chemistry parameter (hematology values, liver and kidney functioning, blood minerals and lipids) measured after 4 weeks of intake

Values are expressed as mean (± SD) and were compared with paired two-sample t-tests when the normality assumption was met. If not, data are represented as median [IQR] and were compared with Wilcoxon signed rank tests. p-values are not corrected for multiple testing

Type	Parameter	ΔProbiotic (end - start)	ΔPlacebo (end - start)	p-value
Bowel habits	Number of stools per day (Daily)	0 [-0.04,0.43]	0 [-0.19,0.28]	0.517
Bowel habits	Bristol stool score (1=separate hard lumps, 7=watery stool) (Daily)	0.03 [-0.26,0.36]	0.1 [-0.25,0.42]	0.885
Bowel habits	Abdominal pain (0=no, 100=whole day) (Daily)	0 [-0.25,2]	0 [-1,2.25]	0.59
Bowel habits	Severity abdominal pain (0=no, 100=unbearable) (Daily)	0 [-0.25,1.25]	0 [-1.25,1.25]	0.736
Bowel habits	Severity bloating (0=no, 100=unbearable) (Daily)	0 [-3,2.25]	0 [-3.25,0.25]	0.761
Bowel habits	Stool consistency (0=watery, 100=very hard) (Daily)	0.64 (± 13.57)	-1.71 (± 8.8)	0.357
Bowel habits	Influence abdominal complaints on life (0=no, 100= completely) (Daily)	0 [0,3]	0 [-0.25,2]	0.569
Bowel habits	Abdominal pain: Number of days (Weekly)	0 [0,0]	0 [-0.25,0]	0.806
Bowel habits	Abdominal pain: Severity (0=no, 100=unbearable) (Weekly)	0 [0,1.25]	0 [-4.25,5.25]	0.508
Bowel habits	Abdominal pain: Severity worst period (0=no, 100=unbearable) (Weekly)	0 [-2,0.25]	0 [-3,2]	0.251
Bowel habits	Bloating: Number of days (Weekly)	0 [0,1]	0 [-0.25,0]	0.168
Bowel habits	Bloating: Severity (0=no, 100=unbearable) (Weekly)	0 [0,2]	0 [-5.75,0.25]	0.862
Bowel habits	Bloating: Severity worst period (0=no, 100=unbearable) (Weekly)	0 [-2.25,3.75]	0 [-8,3.25]	0.676
Bowel habits	Stool: Watery (number of days) (Weekly)	0 [-0.25,0]	0 [0,0]	0.497
Bowel habits	Stool: Hard lumps (number of days) (Weekly)	0 [0,0]	0 [0,0]	0.564
Bowel habits	Stool: Hurry to reach toilet (number of days) (Weekly)	0 [0,0]	0 [0,1]	0.869
Bowel habits	Stool: Difficult straining (number of days) (Weekly)	0 [0,0]	0 [0,0]	0.782
Bowel habits	Stool: Incomplete defecation feelings (number of days) (Weekly)	0 [0,0]	0 [0,0]	0.957
Bowel habits	Stool: Severity stool problems (0=no, 100=unbearable) (Weekly)	0 [-3.25,2]	0 [-0.75,8.5]	0.761

Bowel habits	General: Abdominal complaints influenced life (number of days) (Weekly)	0 [0,0]	0 [0,0.25]	0.593
Bowel habits	General: Severity abdominal pain (0=no, 100=unbearable) (Weekly)	0 [-2.25,3.25]	0 [-0.75,6.5]	0.386
Bowel habits	General: Abdominal complaints (0=no, 100=unbearable)	0 [-1.75,2.25]	0 [0,5.25]	0.503
Haematological parameters	Haemoglobin [g/dL]	0.05 (± 0.62)	-0.29 (± 0.68)	0.065
Haematological parameters	Haematocrit	0 [-0.01,0.01]	-0.01 [-0.02,0.01]	0.119
Haematological parameters	Red blood cell (RBC) count [10^12/L]	0.01 (± 0.18)	-0.06 (± 0.18)	0.147
Haematological parameters	Mean corpuscular volume (MCV) [fL]	0.03 (± 1.07)	0.17 (± 0.95)	0.64
Haematological parameters	Mean content of haemoglobin (MCH) [pg]	0 [-0.33,0.4]	0 [-0.5,0.1]	0.191
Haematological parameters	Mean corpuscular haemoglobin concentration (MCHC) [g/dL]	0 [-0.25,0.5]	-0.2 [-0.53,0.1]	0.183
Haematological parameters	Random distribution of RBC weight (RDW) [%]	0 [-0.2,0.1]	0 [-0.23,0.2]	0.709
Haematological parameters	White blood cell count [10^9/L]	0.32 [-0.28,0.65]	-0.35 [-1.08,0.3]	0.104
Haematological parameters	Platelet count [10^9/L]	4.04 (± 21.77)	0.14 (± 20.68)	0.465
Haematological parameters	Mean platelet volume (MPV) [fL]	0.10 (± 0.27)	0.03 (± 0.38)	0.45
Liver and kidney function parameters	Albumin [g/L]	0.07 (± 2.21)	-1.35 (± 2.58)	0.041 (1)
Liver and kidney function parameters	Alkaline phosphatases [U/L]	1.43 (± 5.86)	0.32 (± 7.55)	0.569
Liver and kidney function parameters	Alanine aminotransferase (ALT) [U/L]	0 [-2.25,3]	-1 [-3,1.25]	0.914
Liver and kidney function parameters	Aspartate aminotransferase (AST) [U/L]	0 [-1.5,2.5]	-0.5 [-2,3]	0.809
Liver and kidney function parameters	Bilirubin [mg/dL]	0.02 [-0.07,0.13]	0.01 [-0.16,0.07]	0.349
Liver and kidney function parameters	Creatine kinase [U/L]	2.5 [-7.5,34.75]	1 [-26.5, 24]	0.35
Liver and kidney function parameters	Creatinine [mg/dL]	0.02 (± 0.06)	-0.01 (± 0.08)	0.185
Liver and kidney function parameters	Gamma glutamyl transpeptidase (GT) [U/L]	0.5 [-1,1.25]	-0.5 [-2,1]	0.579
Liver and kidney function parameters	Glucose [mg/dL]	-0.71 (± 6.2)	-0.04 (± 5.23)	0.66
Liver and kidney function parameters	Lactate dehydrogenase (LDH) [U/L]	10.5 [0.5,17.75]	6.5 [-2.75,25.75]	0.741
Liver and kidney function parameters	Total protein [g/L]	0.71 (± 3.34)	-1.07 (± 3.03)	0.044 (2)

Liver and kidney function parameters	Urea [mg/dL]	0.79 (± 4.55)	-1.5 (± 6.76)	0.148
Blood minerals	Bicarbonate [mM]	0.14 (± 3.23)	-0.13 (± 2.45)	0.73
Blood minerals	Calcium [mM]	-0.02 (± 0.07)	-0.06 (± 0.06)	0.008 (3)
Blood minerals	Chloride [mM]	-0.62 (± 1.66)	0.01 (± 1.79)	0.22
Blood minerals	Iron [µg/dL]	9.82 (± 41.46)	-11.04 (± 62.18)	0.154
Blood minerals	Magnesium [mM]	0.01 (± 0.03)	0.01 (± 0.05)	0.899
Blood minerals	Potassium [mM]	-0.09 [-0.32,0.13]	-0.05 [-0.23-0.08]	0.3
Blood minerals	Sodium [mM]	-0.19 (± 1.39)	0.1 (± 1.93)	0.513
Blood lipids	Triglycerides [mg/dL]	-0.11 (± 28.52)	-4.14 (± 34.01)	0.585
Blood lipids	Cholesterol [mg/dL]	2.21 (± 13.26)	0.25 (± 21.72)	0.683
Blood lipids	High-density lipoprotein (HDL) cholesterol [mg/dL]	2 [-1.25,4]	2 [-3.25,4.25]	0.484
Blood lipids	Low-density lipoprotein (LDL) cholesterol [mg/dL]	-1.36 (± 10.13)	-4.18 (± 18.79)	0.503

(1) Only one subject deviated from the normal range (35-52 g/L; 52.4 g/L), and this was at the end of the placebo

(2) No subject deviated from the normal range (70-150 g/L)

(3) Only one subject deviated from the normal range (2.15-2.55 mM; 2.6 mM), and this was at the start of the placebo intervention period
