# Supplementary material for: Butyrate Producers as Potential Next-Generation Probiotics: Safety Assessment of the Administration of Butyricicoccus pullicaecorum to Healthy Volunteers
Source: mSystems. 2018 Nov 6;3(6):e00094-18. doi: 10.1128/mSystems.00094-18 (PMC6222043; doi:10.1128/mSystems.00094-18)
Supplement: TABLE S2 [file sys006182288st2.pdf]

Supplemental Table S2: Differences in relative abundances of microbial genera after probiotic administration (end - start) compared to placebo (end - start) (Wilcoxon signed rank tests)

Genus	Z.statistic	effect.size.r	p-value	FDR
Paraprevotella	2.64	0.35	6.81E-03	4.02E-01
Anaerostipes	-2.37	-0.32	1.60E-02	4.73E-01
Blautia	-2.10	-0.28	3.46E-02	5.48E-01
Odoribacter	2.07	0.28	3.71E-02	5.48E-01
Coprobacter	-1.79	-0.24	7.42E-02	6.97E-01
Dorea	-1.67	-0.22	9.65E-02	6.97E-01
Terrisporobacter	-1.57	-0.21	1.24E-01	6.97E-01
Clostridium_XVIII	-1.56	-0.21	1.21E-01	6.97E-01
Turicibacter	-1.52	-0.20	1.35E-01	6.97E-01
EscherichiaShigella	-1.46	-0.20	1.50E-01	6.97E-01
Coprococcus	-1.44	-0.19	1.54E-01	6.97E-01
Senegalimassilia	1.75	0.23	8.30E-02	6.97E-01
Butyricimonas	1.83	0.24	6.74E-02	6.97E-01
Clostridium_sensu_stricto	-1.22	-0.16	2.31E-01	7.90E-01
Intestinimonas	-1.19	-0.16	2.42E-01	7.90E-01
Bilophila	-1.10	-0.15	2.79E-01	7.90E-01
Ruminococcus2	-1.06	-0.14	3.03E-01	7.90E-01
Megasphaera	-0.95	-0.13	3.57E-01	7.90E-01
Butyricicoccus	-0.93	-0.12	3.63E-01	7.90E-01
Sutterella	-0.91	-0.12	3.71E-01	7.90E-01
Bacteroides	-0.91	-0.12	3.75E-01	7.90E-01
Prevotella	0.99	0.13	3.34E-01	7.90E-01
Alloprevotella	1.05	0.14	3.03E-01	7.90E-01
Enterococcus	1.11	0.15	3.44E-01	7.90E-01
Fusobacterium	1.13	0.15	2.66E-01	7.90E-01
Methanobrevibacter	1.13	0.15	2.66E-01	7.90E-01
Akkermansia	1.17	0.16	2.49E-01	7.90E-01
Slackia	1.20	0.16	2.43E-01	7.90E-01
Parasutterella	-0.83	-0.11	4.17E-01	7.97E-01
Dialister	-0.74	-0.10	4.69E-01	7.97E-01
Fusicatenibacter	-0.74	-0.10	4.69E-01	7.97E-01
Parabacteroides	-0.71	-0.10	4.91E-01	7.97E-01
Intestinibacter	-0.70	-0.09	4.94E-01	7.97E-01
Clostridium_XlVa	-0.69	-0.09	5.03E-01	7.97E-01
Clostridium_IV	-0.67	-0.09	5.13E-01	7.97E-01
Desulfovibrio	0.74	0.10	4.75E-01	7.97E-01
Streptococcus	0.74	0.10	4.68E-01	7.97E-01
Holdemanella	0.83	0.11	4.14E-01	7.97E-01
Megamonas	0.58	0.08	5.73E-01	8.25E-01
Faecalicoccus	0.60	0.08	5.63E-01	8.25E-01

Faecalibacterium	0.63	0.08	5.46E-01	8.25E-01
Gemmiger	-0.47	-0.06	6.48E-01	8.33E-01
Lactobacillus	-0.47	-0.06	6.60E-01	8.33E-01
Bifidobacterium	0.46	0.06	6.63E-01	8.33E-01
Phascolarctobacterium	0.46	0.06	6.58E-01	8.33E-01
Lactococcus	0.49	0.07	6.56E-01	8.33E-01
Barnesiella	0.51	0.07	6.18E-01	8.33E-01
Acidaminococcus	0.37	0.05	7.22E-01	8.88E-01
Romboutsia	-0.34	-0.05	7.41E-01	8.93E-01
Alistipes	-0.30	-0.04	7.74E-01	9.07E-01
Ruminococcus	0.29	0.04	7.84E-01	9.07E-01
Flavonifractor	-0.22	-0.03	8.38E-01	9.35E-01
Collinsella	-0.20	-0.03	8.50E-01	9.35E-01
Clostridium_XlVb	-0.19	-0.03	8.59E-01	9.35E-01
Veillonella	-0.17	-0.02	8.72E-01	9.35E-01
Victivallis	0.12	0.02	9.16E-01	9.65E-01
Oscillibacter	0.09	0.01	9.39E-01	9.71E-01
Roseburia	0.00E+00	0.00E+00	1.00E+00	1.00E+00
Sporobacter	0.01	1.96E-03	1.00E+00	1.00E+00
